# Low Use of Skilled Attendants' Delivery Services in Rural Kenya

**Published:** 2006-12

**Authors:** Kristen Cotter, Mark Hawken, Marleen Temmerman

**Affiliations:** ^1^ University of Pittsburgh, Pittsburgh, Pennsylvania, Tulane University, New Orleans, Louisiana, and Women and Infants Hospital, Providence, Rhode Island, USA; ^2^ International Centre for Reproductive Health, Mombasa, Kenya; ^3^ Department of Obstetrics/Gynecology, Faculty of Medicine and Health Sciences, Ghent University, Ghent, Belgium, and International Centre for Reproductive Health, Ghent

**Keywords:** Delivery, Obstetric care, Skilled birth attendants, Health facilities, Births, Maternity, Kenya

## Abstract

The aim of the study was to estimate the use of skilled attendants’ delivery services among users of antenatal care and the coverage of skilled attendants’ delivery services in the general population in Kikoneni location, Kenya. Data collected from the registers at the Kikoneni Health Centre (KHC) from March 2001 through March 2003 were retrospectively reviewed. Antenatal care attendance, deliveries by skilled attendants, and the percentage of antenatal care attendees who delivered in a healthcare facility were assessed. Deliveries at the KHC were compared with expected births in the population to estimate the coverage of deliveries assisted by skilled attendants in the community. Of 994 women who attended the antenatal care clinic, 74 (7.4%) presented for delivery services. 5.4% of expected births in the population occurred in health facilities. The coverage of deliveries assisted by skilled attendants was far below the national and international goals. The use of institutional delivery services was very low even among antenatal care attendees. Targeted programmatic efforts are necessary to increase skilled attendant-assisted births, with the ultimate goal of reducing maternal mortality.

## INTRODUCTION

In 1997—10 years after launching the Safe Motherhood Initiative—the Inter-Agency Group for Safe Motherhood established an action agendum for reducing maternal mortality. It concluded that “the single most critical intervention for safe motherhood is to ensure that a health worker with midwifery skills is present at every birth” (1).

Meanwhile, antenatal care has long been a traditional component of maternal care in both developed and developing countries, despite the lack of conclusive evidence that antenatal care reduces maternal mortality (2–4). While antenatal care in and of itself may have some benefit for both infants and mothers, it is also valued for serving as an entry point into the healthcare system, which may lead a woman to skilled attendant-assisted delivery at the culmination of her pregnancy (3).

Especially in sub-Saharan Africa, many more women attend antenatal care clinics than seek skilled attendants’ delivery services, although the magnitude of this differential varies from country to country and regionally within border areas of countries (5). This means that even among women who have formal interactions with the healthcare system through antenatal care-seeking, a significant subset still delivers without adequate obstetric care.

In early 2003, we conducted a baseline safe-motherhood needs assessment in Kikoneni and Dzombo locations in Kwale district of Kenya; these two locations represent a rural area, about a two-hour drive south of the city of Mombasa. The complete assessment included information on facilities, supplies, human resources, practices of traditional birth attendants, and experiences of antenatal care clients. Here, we report on the use of antenatal care and skilled attendance at delivery, as recorded in the official data registers at the Kikoneni Health Centre (KHC) for the previous two years.

The aim of this component of the study was to estimate the use of skilled attendants’ delivery services among users of antenatal care and the coverage of skilled attendants’ delivery services in the general population of Kikoneni location, Kenya.

## MATERIALS AND METHODS

The KHC, a public primary healthcare facility run by the Kenyan Ministry of Health, provides basic medical care for a catchment population of 44,647 in Kikoneni and Dzombo locations in Kwale district. A clinical officer-in-charge, four nurses, a laboratory technician, and a public health officer work in the KHC. Services provided by the KHC include curative care, family planning, antenatal care, immunizations, diagnosis and treatment of tuberculosis, and delivery assisted by skilled attendants. The staff also oversees the administration of more basic health services at three public dispensaries within the catchment area.

Coast province, where Kikoneni is located, has among the poorest statistics of all of Kenya. Data of the Kenya Demography and Health Survey 2003 showed that 31.2% of women in Coast province gave birth in a health facility compared to 40.1% nationally. Only one other province ranked lower than Coast province, that is Western province at 28.4%, with other provinces ranging from 32.9% to 66.9% (6).

The KHC is located 29 km from the Msambweni District Hospital, and 19 of the 29 km have unpaved road. Minibus is the only transportation, which passes from Kikoneni to Msambweni but usually only on Tuesday market days, or by bicycle or pushcart. Local residents in the area own few private vehicles, and sometimes emergency transport can be arranged among families and owners of these vehicles.

The KHC nurses who serve as birth attendants are trained in normal delivery care, manual removal of the placenta, repair of episiotomy and laceration, bimanual and speculum examinations, and active management of the third stage of labour (though without using oxytocin). Anti-convulsant drugs, anti-hypertensive drugs, and antibiotics are available, while uterotonics are not included in the current inventory. The health centre contains an adequate supply of gloves and sterile delivery-kits. Obstetric patients in need of comprehensive services, such as blood transfusions or caesarian sections, are referred to Msambweni District Hospital, 29 km away. At the time of the study, the KHC was without any electricity or running water.

For this study, ‘skilled attendant-assisted delivery’ was defined as a delivery that took place at the KHC, or an imminent delivery where the obstetric patient was referred from the maternity ward of the KHC to the Msambweni District Hospital before delivery occurred. Antenatal care attendance was defined as having attended the antenatal clinic at the KHC at least once during pregnancy, as recorded in the Antenatal Care Register of the health centre.

To conduct this retrospective review, data were extracted from the hand-written antenatal care and maternity registers. Permission was obtained from the Kwale District Medical Officer. The registers contained health and demographic information on obstetric patients seen in the clinic during 25 months, inclusive of March 2001 and March 2003. One (KC) of the authors manually extracted data from the log books kept at the health centre and entered the data into an Excel spreadsheet. In the cases of difficulty in interpreting hand-writings in the log books, the entries were reviewed individually with the healthcare providers responsible for entering them, which, in all cases, provided sufficient clarity to use the data. When demographic data were missing, it was recorded as such, and only available information was used in calculations. Data were entered in Excel 8.0 and then imported to EpiInfo 2002. The numbers of skilled attendant-assisted deliveries and antenatal care attendees were computed.

The proportion of antenatal care patients who used skilled attendance at the time of delivery was calculated as follows:

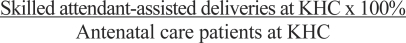


Since the names of the antenatal care patients and patients who delivered at the KHC could not be directly linked, this proportion assumes that all the women who delivered at the KHC are also antenatal care patients at the KHC. This assumption is supported by DHS 2003 data which found that only 11.7% of Kenyan women who received no prenatal care delivered at a healthcare facility (6). Thus, while the assumption is not perfect, it seems reasonable to assume that the great majority of women who do deliver at the KCH are drawn from the pool of women who attend antenatal clinics.

Finally, an estimate of coverage of skilled attendant-assisted deliveries was derived by comparing the recorded skilled attendant-assisted deliveries with expected birth rates for Kikoneni location during the same period. The expected number of births in a 25-month period was 1,373, as derived from the 1999 Kenyan national census data, based on the crude birth rate of 45 per 1,000 population and Kikoneni location's population of 14,647, and adjusted for the 25-month period covered by the study.

Unlike skilled attendant-assisted deliveries, antenatal care is available from a few other sites in the area besides the KHC. Therefore, while the estimates of coverage of skilled attendant-assisted deliveries for the area were calculated using the KHC records, the coverage of antenatal care was not.

## RESULTS

During the 25 recorded months, 994 women presented for antenatal care at the KHC, averaging 43.5 new clients per month. The mean age of patients was 22.4 years, with adolescents, aged less than 20 years, accounting for 33.2% of all new clients. Nulliparous antenatal care patients had a mean age of 18.6 years. The mean gestational age of first presentation for antenatal care was 26.1 weeks, with 44.7% of the clients presenting for their first visit during the third trimester. These results are presented in the table.

**Table.** Demographic data from 994 antenatal care clients

Characteristics	Results	Missing (%)
Mean age (years), all patients	22.4	17.3
Mean age (years), nulliparous patients	18.6	n/a
Adolescents (%), of all patients	33.2	n/a
Mean gestational age (weeks) of first presentation for antenatal care	26.1	40.6
Patients (%) presenting during third trimester	44.7	n/a

n/a=Not applicable

During the same time period, 74 women presented for delivery services. Seven of them were referred to the Msambweni District Hospital for complications, such as antepartum haemorrhage (n=1), eclampsia (n=1), and unspecified causes (n=5). One stillbirth and no maternal or infant deaths occurred during this period.

If it is assumed that all the women who gave birth at the KHC had attended the antenatal clinic there at least once, it can be calculated that 7.4% (74 of 994) of antenatal patients used skilled attendants at delivery.

In the 25-month period when 1,373 births were expected in Kikoneni location, 74 skilled attendant-assisted deliveries were recorded by the KHC, yielding an estimated skilled attendants’ delivery coverage of 5.4%.

## DISCUSSION

This review of the records from the KHC suggests that the current coverage of skilled attendant-assisted delivery falls far below the current Kenyan national average of 40.1% (6) and drastically below the Kenyan national goal of the coverage of 80% by 2010.

The difference between observed skilled attendance at delivery and that obtained from the DHS data can be explained by a number of possibilities. It is possible that the coverage of skilled attendance at delivery in Kikoneni is, indeed, significantly below average even for Coast province, and its very low coverage is balanced by a higher coverage in other areas, so that the mean for the whole province hides Kikoneni's below average numbers. Alternatively, it may be that self-reported population-based data over-estimate outcomes due to social desirability bias, and perhaps outcome measures that directly record attendence at health facilities are closer to accurate measures.

It is also possible that women who attend the antenatal care clinic at the KHC may be travelling to the Msambweni District Hospital for skilled attendant-assisted delivery, thereby artificially deflating our estimates. Given that accessible and affordable transportation is nearly non-existent, it seems unlikely that significant numbers of women use this alternative. The healthcare providers at the KHC, who live onsite and know the community well, and the local community health workers, describe an environment where it is all but unheard of for women to seek a planned delivery at the district hospital without ever having presented to the more local KHC first. Most women instead give birth in their village with the assistance of traditional birth attendants, including many who have received some formal training on peripartum care.

If the coverage rate for skilled attendant-assisted delivery is truly close to the 5.4% calculated here, this region of Kenya ranks just better than Equatorial Guinea, where the coverage of skilled attendant-assisted delivery is 5.0%, ranking it the lowest in the world. There is only one other country on the African continent which ranks below 15%—Ethiopia at 9.8%. The next lowest are Niger at 15.7%, Chad at 16.2%, and Burundi at 19.1% (7). While Kenya as a whole are the best-served area with a coverage of 40.1%, the coverage of skilled attendant-assisted delivery in Kikoneni location is comparable with the least-served areas of the world.

Perhaps, most notable is the low use of skilled attendants’ services among women who use antenatal care. Multiple studies, including DHS data and the Bangladesh Maternal Health Services and Maternal Mortality Survey 2001 (8), suggest that women who use prenatal care are far more likely to use delivery services than those who receive no prenatal care. At the same time, studies have also shown that a much larger percentage of women seek antenatal care than seeking delivery care. This study in Kikoneni again emphasizes that, even among women who intentionally seek out the benefits of antenatal care at a healthcare facility, the key safe motherhood action-message of ensuring ‘skilled attendance at delivery’ has not yet been embraced. Despite the provision of services, the use of skilled attendant-assisted delivery remains minimal. Antenatal care is not yet meeting its potential to serve as an entry into the healthcare system at the time of delivery.

The findings of this study on the coverage of skilled attendants’ delivery services prompted a formal discussion among three community mobilizers in Kikoneni location and employees of the International Centre for Reproductive Health (ICRH)'s Uzazi Bora Programme in January 2006, specifically to address the factors contributing to the low rate of coverage. Open-ended questions were used, and the community mobilizers were prompted to provide case examples of women in their communities to back-up their opinions. Notes were taken, and key points were summarized. The community mobilizers identified the following four reasons why skilled attendant-assisted delivery was so low in the area:

- Logistic barriers, including distance, frequent absence of any available transportation, and cost of transportation when it is available. An organized transportation system is nearly non-existent in the area.
- Lack of sensitization among women regarding the importance of skilled attendance at delivery. The mobilizers describe a sense that, because the previous generation delivered at home, so too will this generation of women. Similarly, since it is only a minority of deliveries that are complicated by life-threatening events, witnessing of normal deliveries in a woman's own life or in that of her friends or relatives reinforces the idea that delivery at home is safe. The healthcare providers at the KHC may not be actively attempting to sensitize women to the importance of delivering with skilled assistance. In exit-interviews conducted as part of the ICRH baseline needs assessment, 35 women were interviewed as they emerged from prenatal care visits at the KHC. Only 17% reported being counselled at any visit of their pregnancy thus far on developing a birth-plan regarding the location of her delivery, and 8.6% reported being told the benefits of delivering in a health facility (9).
- The value women place on delivery by a traditional birth attendant (TBA). The social role played by TBAs in communities is recognized and respected, and therefore, their attendance is highly valued. They provide services that the formal health system does not, including postpartum care in the home. Additionally, a minority of women believe that childbirth-related complications are caused by witchcraft, and TBAs are perceived as better equipped to intervene in these cases.
- The perception that the health facility is a harsh setting for childbirth. One community health worker said, “They leave you alone during your labour pain at the health centre; the TBA stays with you and helps you to deal with the pain.” This perception is a reflection of the chronic under-staffing of the KHC. When a woman is labouring in the maternity ward during the day, the nurse on duty attends not only to her labour, but also to the busy outpatient clinic for children and adults. As a result of the many duties, it is not unusual that a woman under the medical care of the KHC staff spends a large portion of her labour without the nurse being present in the room.

Issues regarding distance from the health centre and access to transportation are dependent on the long-term development of economy and infrastructure. These issues, however, are only part of the problem, and several alternative opportunities for intervention closer to the community and health centre are presented here. It is possible that healthcare providers are failing to adequately use antenatal care as an opportunity to encourage women to deliver at their healthcare facilities. Training healthcare providers to emphasize the importance of clean, safe delivery in reducing maternal mortality may be a key starting point. Additionally, training community health workers may deliver this message closer to the community level and could be helpful in addressing the cultural norms and gender issues that influence decision-making. The installation of running water and possibly electricity at the KHC could result in a more comfortable and sanitary facility that is not only better-equipped for its purposes, but is also perceived as being capable of providing care beyond what is offered in the home. Furthermore, efforts to improve the experience of women who choose to deliver at the KHC must be addressed. Given that verbal reports of women's birthing experiences are likely to reach many people in the community, the health centre staff must prioritizes the emotional needs of labouring patients. Finally, the possibility of allowing TBAs to accompany labouring women in the maternity ward of the KHC should be considered.

In conclusion, the coverage of skilled attendants’ delivery services in Kikoneni location, Kenya, fell far below the national and international goals aimed at reducing maternal mortality. Since antenatal care is used far more frequently than delivery services, the population attending antenatal clinics represents a source of great potential for efforts to increase the percentage of skilled attendant-assisted deliveries. These women have already demonstrated some acceptance of the healthcare system by their antenatal clinic attendance and present a readily-accessible opportunity for one-on-one counselling on the benefits of delivering at a health facility. The healthcare providers should take the full advantage of this opportunity.

## ACKNOWLEDGEMENTS

The study was partly funded by the European Commission as part of the Uzazi Bora Programme implemented by the International Centre for Reproductive Health in Mombasa, Kenya.
